# Comparison of apolipoprotein-A1 levels between paroxysmal atrial fibrillation patients and healthy subjects

**DOI:** 10.34172/jcvtr.2020.23

**Published:** 2020-05-17

**Authors:** Tufan Çınar, Veysel Ozan Tanık, Kadir Gürkan

**Affiliations:** ^1^Health Sciences University, Sultan Abdülhamid Han Training and Research Hospital, Department of Cardiology, Istanbul, Turkey; ^2^Ankara Dışkapı Yıldırım Beyazıt Training and Research Hospital, Department of Cardiology, Ankara, Turkey; ^3^Health Sciences University, Siyami Ersek Training and Research Hospital, Department of Cardiology, Istanbul, Turkey

**Keywords:** Paroxysmal Atrial Fibrillation, Apolipoprotein-A1, Inflammation

## Abstract

***Introduction:*** Studies found that the inflammation plays a key role in the pathogenesis of paroxysmal atrial fibrillation (PAF). It is well-known that apolipoprotein-A1 (Apo-A1) demonstrates antiinflammatory and anti-oxidant properties in a healthy physiological system. In the present study, we aimed to determine whether there is any difference of Apo-A1 levels in patients with PAF and healthy subjects.

***Methods:*** In this prospective cohort study, we enrolled a total of 35 PAF patients and 34 comparable healthy participants. Apo-A1 levels were measured from each subject using an immunophelometric method. All enrolled subjects were followed-up for one year during the study period. ***Results:*** Serum high-sensitivity C-reactive protein (hs-CRP) levels were statistically higher in PAF patients compared to healthy subjects (1.54±1.99 vs. 1.06±2.01, *P* = 0.016, respectively). Of note, patients with PAF had lower Apo-A1 levels (1.84±0.74 vs. 2.55±0.44, *P* = 0.001, respectively). There was no statistical difference between the groups in terms of apolipoprotein-B levels (1.08±0.36 vs. 0.99±0.38, *P* = 0.339, respectively). We did not find any correlation between Apo-A1 levels and PAF attacks in the study.

***Conclusion:*** The main finding of this study was that Apo-A1 levels were significantly lower in PAF patients compared to healthy participants. Based on our results, we considered that Apo-A1 may have a key role in the pathogenesis of PAF.

## Introduction

Atrial fibrillation (AF) is a frequent supraventricular arrhythmia characterized by an irregular, disorganized, and rapid atrial activation.^1^ Paroxysmal atrial fibrillation (PAF), which is a subtype of AF, is usually in an intermittent nature and terminates spontaneously or with intervention in less than 7 days. Recent studies have found that the inflammation might have a key role in the initiation and perpetuation of PAF.^2,3^ The elevation of some inflammatory markers, such as high-sensitivity C-reactive protein (hs-CRP) and interleukin-6, have been found in patients with PAF compared to healthy participants.^4,5^ Furthermore, higher hs-CRP levels have been found to be associated with longer AF duration in a previous study.^6^ In addition to the inflammation, oxidative stress is shown to be involved in the pathogenesis of PAF.^7^

Apolipoprotein-A1 (Apo-A1), which is the major apolipoprotein of high-density lipoprotein (HDL), seems to confer a major protection role against atherosclerosis development in the human body. Besides this important role, Apo-A1 has some anti-inflammatory and anti-oxidant properties, which can exert these affects through relevant enzymes.^8,9^ As the inflammatory status and oxidative stress are involved in the pathophysiology AF, we considered that Apo-A1 levels might be lower among PAF patients. Based on this data, the present study aimed to determine whether there is any difference of Apo-A1 levels in patients with PAF and healthy subjects.

## Materials and Methods

### Data collection

In this prospective, cross-sectional study, we enrolled PAF patients seen in a tertiary heart center emergency department between July 2012 and May 2013. The enrolled patients’ age was between 18 and 60 years. The patients with chronic diseases, such as hypertension, diabetes mellitus, coronary artery disease, congestive heart failure, etc., were excluded from the study. Finally, the study cohort was consisted of a total of 35 PAF patients. The control cohort was composed of 34 age-, sex-, and body mass index (BMI)-matched healthy subjects.

AF was diagnosed with a rapid and irregular heart rhythm without detecting P waves, which was present on the electrocardiography (ECG) taken during palpitation. In patients in whom AF did not terminate spontaneously, normal sinus rhythm was achieved either with medical or electrical cardioversion. Following the restoration of normal sinus rhythm, either beta-blocker or calcum channel blocker or antiarrhythmic were prescribed to prevent AF attack. All patients were followed-up for AF attacks during 1 year study period. The follow-up of each participant was performed using either outpatient visits or telephone interviews.

### Echocardiographic assessment

A trained investigator performed an echocardiographic examination within 30 days following AF attack. The left ventricular end-diastolic diameter (LV-dd), LV end-systolic diameter (LV-sd), LV posterior wall thickness (LV-pw) and inter-ventricular septal thickness (LV-sw) as well as the left atrium (LA) end-systolic dimension were measured using the M-mode method on the parasternal long-axis view. The Simpson’s method was used to measure the LV ejection fraction (LV-ef).

### Laboratory analysis

The blood samples of all patients were collected following 8-12 hours overnight fasting. The standard laboratory methods were used to analyze blood samples for glucose, hemoglobin, creatinine, triglyceride (TG), very low density lipoprotein (VLDL), total cholesterol (TC), HDL, and low-density lipoprotein (LDL) levels.

### Apolipoprotein analysis

First, we collected a 5 ml of blood sample in a dried tube. Then, we centrifuged it at 1000 rpm for at least ten minutes in order to obtain serum sample. After that, we stored all serum samples at -80^°^C until the analysis. Immunophelometric method (Nephstar, Apo-A1 kit) was used to measure serum Apo-A1 level.

### Statistical analysis

All statistical analyses were conducted using Number Cruncher Statistical System 2007 and Power Analysis and Sample Size 2008 statistical software (Utah, USA). The Shapiro-Wilk’s test was used for testing data normality. The mean ± standard deviation was used to express the continuous variables. The student *t* test was used to compare the continuous variables with normal distribution, whereas the Mann-Whitney U was used to compare the continuous variables without normal distribution. In order to compare the qualitative data, either Fisher’s exact test or Yates continuity correction test were performed. Pearson correlation analysis was performed to assess correlation for variables showing normal distribution. On the other hand, Spearmen’s correlation analysis was preferred to assess correlation for variables without normal distribution. It was considered statistically significant when a *P* value was less than 0.05.

## Results

Table 1 is a presentation of baseline demographic features of all enrolled subjects included in the study. The study cohort mean age was 44.52 ± 7.32 years. A total of 48 (69.6%) of the patients were male and 21 of them (30.4%) were female. Both groups were indifferent in terms of demographic characteristics, including age, sex, BMI, etc. (*P* > 0.05 for each). In the study, the mean number of AF attacks was 3.60 ± 3.63 episodes and the mean AF duration was 13.17 ± 10.22 hours.

**Table 1:** The comparison of demographic characteristics of patients with paroxysmal atrial fibrillation and control group

	**Total (n=69)**	**Control (n=34)**	**Patients (n=35)**	***P*** **value**
	**Mean ± SD**	**Mean ± SD**	**Mean ± SD**	
Age, years	44.52±7.32	45.76±5.18	43.31±8.84	0.164^a^
BMI, kg/BSA	26.45±3.51	26.46±3.32	26.44±3.73	0.985^a^
Female, n (%)	21 (30.4)	10 (29.4)	11 (31.4)	1.000^b^
Male, n (%)	48 (69.6)	24 (70.6)	24 (68.6)	1.000^b^
Smokers, n (%)	9 (13)	4 (11.8)	5 (14.3)	1.000^c^
Alcohol usage, n (%)	2 (2.9)	1 (2.9)	1 (2.9)	1.000^c^
Obesity, n (%)	13 (18.8)	6 (17.6)	7 (20)	1.000^b^

Abbreviations: BMI; body mass index, BSA; body surface area, AF; atrial fibrillation. ^a^Student*t* test, ^b^Yates continuity correction, ^c^Fisher’s exact test.

Echocardiographic findings of each subject were displayed in Table 2. LV-ef, LV-dd, LV-sw, and A wave velocity were not different between the groups (*P* > 0.05 for each). On the other hand, the LA dimension was higher and E wave velocity was lower in PAF patients (*P* = 0.02 and *P* = 0.07, respectively).

**Table 2:** Echocardiographic parameters of all participants

	**Total**	**Control (n=34)**	**Patients (n=35)**	***P*** **value**
	**Mean ± SD**	**Mean ± SD**	**Mean ± SD**	
LVEF, %	61.75±2.47	61.88±2.36	61.63±2.6	0.673^a^
LVSD, mm	27.64±4.02	26.44±2.58	28.80±4.79	0.014*^a^
LVDD, mm	46.90±3.69	46.38±2.81	47.4±4.36	0.252^a^
LAP, mm	34.30±4.38	32.68±3.75	35.89±4.42	0.002**^a^
PW, cm	1.05±0.97	0.92±0.05	0.94±0.06	0.060^b^
IVS, cm	0.92±0.07	0.91±0.06	0.94±0.08	0.078^a^
E wave velocity	0.85±0.13	0.89±0.1	0.81±0.15	0.007**^a^
A wave velocity	0.50±0.10	0.48±0.11	0.52±0.09	0.102^a^

Abbreviations: LVEF; left ventricular ejection fraction, LVSD; left ventricular systolic dimension, LVDD; left ventricular diastolic dimension, LAP; left atrial size, IVS; interventricular septum. ^a^Student *t* test, ^b^Mann-Whitney U test, * *P*  < 0.05, ** *P*  < 0.01.

Comparison of laboratory findings of both groups are demonstrated in Table 3. In terms of lipid profile, only VLDL and TG levels were significantly different between the groups (*P* < 0.05 for each). The other lipid parameters were similar both in PAF patients and healthy subjects (*P* > 0.05 for each). Patients with PAF had significantly higher hs-CRP levels than healthy subjects (1.54 ± 1.99 vs. 1.06±2.01, *P* = 0.016, respectively). Of note, patients with PAF had lower Apo-A1 levels (1.84 ± 0.74 vs. 2.55 ± 0.44, *P* = 0.001, respectively). There was no statistical difference between the groups in terms of apolipoprotein-B levels (1.08 ± 0.36 vs. 0.99 ± 0.38, *P* = 0.339, respectively).

**Table 3:** The comparison of biochemical and hematological measurements between paroxysmal AF and control group

	**Total**	**Control (n=34)**	**Patients (n=35)**	***P*** **value**
	**Mean ±SD**	**Mean ± SD**	**Mean ± SD**	
HbA1c, mg/dL	5.69±0.33	5.62±0.36	5.75±0.30	0.123^a^
LDL, mg/dL	122.07±28.41	122.21±28.25	121.94±28.98	0.970^a^
VLDL, mg/dL	26.13±13.48	21.73±11.54	30.40±13.99	0.007**^b^
TC, mg/dL	199.78±36.64	198.21±38.22	201.31±35.53	0.727^a^
HDL, mg/dL	50.30±11.50	52.74±10.24	47.94±12.30	0.084^a^
TG, mg/dL	129.30±64.22	111.91±59.48	146.2±64.95	0.025*
BUN, mmol/L	14.39±3.07	15.12±2.96	13.69±3.06	0.052^a^
Creatinine, mg/dL	0.84±0.15	0.82±0.13	0.85±0.16	0.347^a^
AST, U/L	24.13±8.16	23.35±8.46	24.89±7.90	0.439^a^
ALT, U/L	29.2±23.57	26.5±23.28	31.83±23.89	0.003**^b^
Glucose, mg/dL	93.35±8.85	92.85±8.05	93.83±9.66	0.651^a^
hs-CRP, mg/dL	2.08±6.65	1.06±2.01	1.54±1.99	0.016*^b^
Apo-A1, mg/dL	2.19±0.70	2.55±0.44	1.84±0.74	0.001**^a^
Apo-B, mg/dL	1.03±0,37	1.08±0.36	0.99±0,38	0.339^a^
Apo-B/ Apo-A1	0.51±0.21	0.42±0.12	0.59±0.24	0.002**^b^
Hemoglobin, g/dL	14.28±1.43	14.18±1.31	14.37±1.54	0.597^a^
Hematocrit, %	42.85±4.08	42.45±3.78	43.23±4.39	0.433^a^
WBC, x 10^3^µL	6.58±1.73	6.63±1.87	6.53±1.6	0.821^a^
MPV, fL	8.93±0.85	8.79±0.82	9.08±0.87	0.160^a^

Abbreviations: LDL; low-density lipoprotein, VLDL; very-low density lipoprotein, HDL; high-density lipoprotein, TC; total cholesterol, TG; triglyceride, MPV; mean platelet volume, hs-CRP; high-sensitivity C-reactive protein, HCT; hematocrit, WBC; white blood cell, HbA_1c_; hemoglobin A1c, BUN; blood urea nitrogen, AST; aspartate aminotransferase, ALT; alanine aminotransferase. ^a^Student *t* test, ^b^Mann-Whitney U test, * *P* < 0.05, ** *P* < 0.01.

In a correlation analysis, there was no correlation between Apo-A1 levels and AF attacks, AF duration, and the LA diameter (*r* = -0.166, *P* = 0.235, *r* : -0.070, *P* = 0.691, and*r* = -0.136, *P* = 0.436, respectively).

## Discussion

In this observational and prospective study, we found the following: I-) PAF patients had lower Apo-A1 levels than healthy participants II-) there was no any relation between Apo-A1 levels and AF attacks, AF duration, and the LA diameter in patients with PAF III-) this study result might provide evidence that Apo-A1 levels might have an important role in the pathophysiology of AF.

Recent experimental and clinical studies have demonstrated the importance of inflammation in the pathogenesis of AF.^10,11^ The biopsy samples that were obtained from AF patients undergoing cardiac surgery revealed the presence of inflammatory infiltrates and fibrosis in the atrium.^10^ Additionally, studies have found that both hs-CRP and interleukin-6 levels were significantly elevated in patients with PAF.^4,5^ Similar to this published clinical studies, PAF patients had higher hs-CRP levels than healthy subjects in our study. Besides baseline elevated inflammatory status, oxidative stress and their markers have been linked to increased risk of AF occurrence. Experimental animal studies showed that oxidative modification of lipoprotein might cause a cardiac myocyte damage and electrophysiologic alterations via disrupting transmembrane ion currents.^12,13^ Hence, it was considered that decreased anti-oxidative functions as result of elevated lipid oxidation might be present in AF patients. Similar to these experimental studies, in a recent study, Samman et al reported that increased oxidative stress is related with more prevalent and incident AF.^14^

Lipid profiles in AF patients were investigated in a previous study.^15^ It was found that PAF patients had similar HDL and LDL levels. Similarly, a consistent finding was found in our study regarding to these lipid parameters. These study findings may indicate that some other pathological mechanisms are involved in the AF development.

Apo-A1, which is the main apolipoprotein of HDL, has some important anti-inflammatory and anti-oxidant properties in a healthy physiological system. Prior studies have showed that decreased Apo-A1 levels are closely associated with increased risk of coronary artery disease, stroke, and acute coronary syndrome.^16-18^ Besides that, in a small study included only 11 female patients, Kim et al demonstrated that patients with PAF had an approximately 30% lower expression of Apo-A-1 compared to the healthy subjects.^15^ The present study, which was conducted in a larger sample size compared to the abovementioned study, also found that not only female PAF patients but also male PAF patients had lower Apo-A1 levels than healthy subjects. However, although Apo-A1 were decreased in such patients, we could not find any relation between Apo-A1 levels and AF attacks and duration. As the possible explanations of the study findings, we considered that patients with lower Apo-A1 levels may have a decreased anti-oxidant and anti-inflammatory capacity, which may result in a higher risk of AF. Therefore, further prospective studies with larger population are needed to assess the exact role of Apo-A1 levels in PAF patients.

### Limitations of the study

Our study had following limitations. First, the study had a relatively small number of patients. Second, we only enrolled patients with PAF. Hence, the study results might not be generalized to all AF patients. Third, because the follow-up of each participant was performed using either outpatients visits or telephone interviews, we might miss some AF attacks in the study. Finally, we could not repeat Apo-A1 measurements during follow-up period.

## Conclusion

The main finding of this study was that Apo-A1 levels were significantly lower in PAF patients compared to healthy participants. Based on our results, we considered that Apo-A1 may have a key role in the pathogenesis of PAF.

## Competing interests

The authors declare that they have no conflict of interest.

## Ethical approval

All procedures were in accordance with the ethical standards of the local committee and with the 1975 Declaration of Helsinki, as revised 2008. The design of the present was reviewed and approved by the Institutional Local Committee. An informed consent was obtained from each subject included in the study.

## Acknowledgments

This research article was presented as an oral presentation in the 18th International Eastern Mediterranean Family Medicine Congress, on 25th-28th April 2019.

## References

[1] Go AS, Hylek EM, Phillips KA, Chang Y, Henault LE, Selby JV (2001). Prevalence of diagnosed atrial fibrillation in adults: national implications for rhythm management and stroke prevention: the Anticoagulation and Risk Factors in Atrial Fibrillation (ATRIA) Study. JAMA.

[2] Issac TT, Dokainish H, Lakkis NM (2007). Role of inflammation in initiation and perpetuation of atrial fibrillation: a systematic review of the published data. J Am Coll Cardiol.

[3] Van Wagoner DR (2008). Oxidative stress and inflammation in atrial fibrillation: role in pathogenesis and potential as a therapeutic target. J Cardiovasc Pharmacol.

[4] Sata N, Hamada N, Horinouchi T, Amitani S, Yamashita T, Moriyama Y (2004). C-reactive protein and atrial fibrillation. Is inflammation a consequence or a cause of atrial fibrillation? Jpn Heart J.

[5] Psychari SN, Apostolou TS, Sinos L, Hamodraka E, Liakos G, Kremastinos DT (2005). Relation of elevated C-reactive protein and interleukin-6 levels to left atrial size and duration of episodes in patients with atrial fibrillation. Am J Cardiol.

[6] Watanabe T, Takeishi Y, Hirono O, Itoh M, Matsui M, Nakamura K (2005). C-reactive protein elevation predicts the occurrence of atrial structural remodeling in patients with paroxysmal atrial fibrillation. Heart Vessels.

[7] Korantzopoulos P, Kolettis TM, Kountouris E, Dimitroula V, Karanikis P, Pappa E (2005). Oral vitamin C administration reduces early recurrence rates after electrical cardioversion of persistent atrial fibrillation and attenuates associated inflammation. Int J Cardiol.

[8] Barter PJ, Puranik R, Rye KA (2007). New insights into the role of hdl as an anti-inflammatory agent in the prevention of cardiovascular disease. Curr Cardiol Rep.

[9] Du R, Winarsih I, Ho B, Ding JL (2012). Lipid-free apolipoprotein A-I exerts an antioxidative role against cell-free hemoglobin. Am J Clin Exp Immunol.

[10] Cosgrave J, Foley JB, Kelly R, McGovern E, Bennett K, Young V (2005). Perioperative serum inflammatory response and the development of atrial fibrillation after coronary artery bypass surgery. Heart.

[11] Ommen SR, Odell JA, Stanton MS (1997). Atrial arrhythmias after cardiothoracic surgery. N Engl J Med.

[12] Pogwizd SM, Onufer JR, Kramer JB, Sobel BE, Corr PB Induction of delayed after depolarizations and triggered activity in canine Purkinje fibers by lysophosphoglycerides. Circ Res.

[13] Adamantidis MM, Moreau-Guedon L, Martin-Nizard F, SqalliHoussaini H, Duriez P, Fruchart JC Oxidized low density lipoproteins exert arrhythmogenic effects in rabbit Purkinje fibers. Biochem Biophys Res Comm.

[14] Samman Tahhan A, Sandesara PB, Hayek SS, Alkhoder A, Chivukula K, Hammadah M (2017). Association between oxidative stress and atrial fibrillation. Heart Rhythm.

[15] Kim SM, Lee JH, Kim JR, Shin DG, Lee SH, Cho KH (2011). Female patients with atrial fibrillation have increased oxidized and glycated lipoprotein properties and lower apolipoprotein A-I expression in HDL. Int J Mol Med.

[16] Rahim S, Abdullah HM, Ali Y, Khan UI, Ullah W, Shahzad MA (2016). Serum Apo A-1 and Its Role as a Biomarker of Coronary Artery Disease. Cureus.

[17] As S, Sahukar S, Murthy J, Kumar K (2013). A study of serum apolipoprotein A1, apolipoprotein B and lipid profile in stroke. J Clin Diagn Res.

[18] Casillas-Muñoz F, Valle Y, Muñoz-Valle JF, Martínez-Fernández DE, Reynoso-Villalpando GL, Flores-Salinas HE (2018). APOA1 and APOB polymorphisms and apolipoprotein concentrations as biomarkers of risk in acute coronary syndrome: Relationship with lipid-lowering therapy effectiveness. Med Clin (Barc).

